# Oral cancer overexpressed 1 (ORAOV1) regulates cell cycle and apoptosis in cervical cancer HeLa cells

**DOI:** 10.1186/1476-4598-9-20

**Published:** 2010-01-28

**Authors:** Lu Jiang, Xin Zeng, Zhi Wang, Ning Ji, Yu Zhou, Xianting Liu, Qianming Chen

**Affiliations:** 1 State Key Laboratory of Oral Diseases, West China College of Stomatology, Sichuan University, Chengdu, China; 2 Key Laboratory of Bio-resources and Eco-environment of Ministry of Education, Sichuan University, Chengdu, China

## Abstract

**Background:**

Oral Cancer Overexpressed 1 (ORAOV1) is a candidate protooncogene locating on 11q13. Recent studies show that ORAOV1 acts as a primary driving force behind 11q13 gene amplification and plays a functional role in the tumorigenesis in a variety of human squamous cell carcinomas (SCCs). According to the results of molecular cytogenetic methods, 11q13 was characterized to be a high-level and recurrent amplification chromosomal site in cervical cancers. Up till now, the role of ORAOV1 in cervical cancer is unknown. The purpose of this study is to elucidate the function of ORAOV1 in cervical cancer cell growth by studying its roles in HeLa cells using small interfering RNA.

**Results:**

Functional analyses revealed that ORAOV1 was involved in the regulation of HeLa cell growth through its effect on cell cycle and apoptosis. Silence of ORAOV1 in HeLa cells downregulated the expression of Cyclin A, Cyclin B1 and Cdc2, and led to a distinct S cell cycle arrest. Moreover, knockdown of ORAOV1 expression activated both extrinsic and intrinsic apoptotic pathways and led to apoptosis in HeLa cells through its effect on the expression of several apoptosis related proteins such as P53, Bcl-2, Caspase-3, Caspase-8, Caspase-9 and cytochrome c. Interestingly, the expression of Cyclin D1, a pivotal gene for cervical cancer tumorigenesis, was also found to be reduced in ORAOV1 silenced HeLa cells.

**Conclusion:**

Our findings indicate that ORAOV1 has an important role in regulating cell growth of cervical cancer HeLa cells through regulating the cell cycle and apoptosis. Thus, it may be a crucial protooncogene and a novel candidate therapeutic target for cervical cancer.

## Background

Oral Cancer Overexpressed 1 (ORAOV1) is a candidate protooncogene in a variety of human squamous cell carcinomas (SCCs) [1]. According to previous studies, ORAOV1 was first identified by Huang and his colleagues at chromosomal band 11q13 [1]. It was supposed to be a primary driving force behind 11q13 gene amplification and regarded as a candidate oncogene with a role in the development and progression of various human SCCs [1]. In following years, several clinical studies showed that the expression level of ORAOV1 was tightly correlated with prognosis-related clinicopathological parameters and clinical grades in several SCCs such as esophageal squamous cell carcinoma and oral squamous cell carcinoma (OSCC) [2,3]. Further functional studies showed that ORAOV1 may have an important role in the tumorigenesis of OSCC by taking part in the regulation of cell growth and tumor angiogenesis [4]. Therefore, it is suggested that ORAOV1 may be a valuable biological marker in SCCs.

Chromosomal band 11q13 has been proved to be one of the most frequently amplified regions in a variety of SCCs [1], and its rearrangements are regarded to be independent prognostic factors for several SCCs [5-7]. Because of its tight correlation with SCCs, chromosomal band 11q13 is suggested to be one of the most frequent tumor related chromosome regions in SCCs. In cervical cancers, using a combination of molecular cytogenetic methods, 11q13 was also characterized as a high-level and recurrent amplification chromosomal site [8]. Because of significant correlation between 11q13 and cervical cancer, and the important role of ORAOV1 in 11q13 amplification, it is of great interest to determine whether ORAOV1 is also involved in the tumorigenesis of cervical cancer or if it is a candidate protooncogene or a potential therapeutic target in cervical cancers as it is in other kinds of SCCs.

HeLa cells are one of the most representative cervix squamous carcinoma cell lines. Based on the results of comparative genomic hybridization (CGH), HeLa cells have an amplification of 11q13 [9]. We chose HeLa cells to investigate the biological functions of ORAOV1 in cervical cancer tumorigenesis through a loss-of-function study by small interfering RNA (siRNA) [10,11]. In this study, we reported for the first time that silence of ORAOV1 in HeLa cells significantly inhibited cell growth through inducing S-phase cell cycle arrest and apoptosis. Thus, we deduce that ORAOV1 plays a crucial role in cervical cancer tumorigenesis, and may be a novel protooncogene and candidate therapeutic target for cervical cancer.

## Results

### ORAOV1 silencing inhibits cell growth and colony formation ability of HeLa cells in vitro

**Figure 1:**
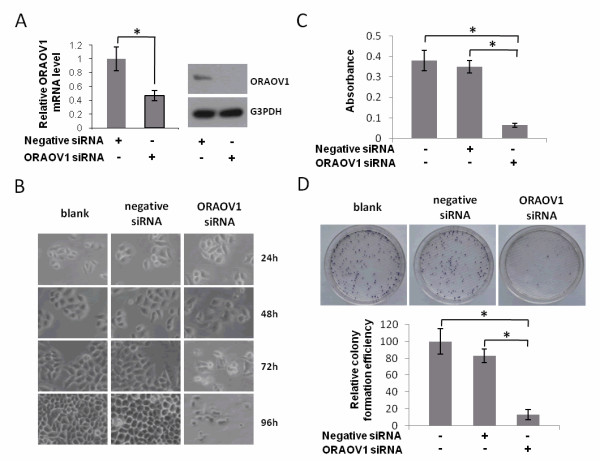
**ORAOV1 silencing inhibits cell growth and colony formation ability in HeLa cells**. (A) The expression levels of ORAOV1 mRNA and ORAOV1 protein in HeLa cells upon transfection with 100 nM ORAOV1 siRNA for 48 hrs were analyzed by real time PCR and Western blot assay respectively. 100 nM non-targeting siRNA-transfected HeLa cells and blank cells were used as controls. Results represent the means ± SE (n = 3). *, *P *< 0.05. (B) The effect of ORAOV1 silencing on HeLa cell growth. (C) Examination the effect of ORAOV1 silencing on HeLa cell proliferation by MTT assay. Results represent the means ± SE (n = 3). *, *P *< 0.001. (D) Test the effect of ORAOV1 silencing on HeLa cell colony formation ability by plate colony assay. The relative colony formation efficiency of the blank cells was set as 1. Each column represents a mean value of triplicate experiments in each group. Data are Mean ± SE, *, *P *< 0.001.

To study the functions of ORAOV1 in HeLa cell growth, we knocked down ORAOV1 in HeLa cells by ORAOV1 siRNA as described in the Materials and Methods section. Our results showed that ORAOV1 was successfully silenced by ORAOV1 siRNA at both the mRNA level and the protein level (Figure 1A). Upon transfection, an apparent suppression of cell growth was observed in ORAOV1 silenced HeLa cells (Figure 1B). At 96 hours after transfection, the viability of the ORAOV1 siRNA transfected HeLa cells decreased by about 80% compared with the controls according to the MTT assay (p < 0.001) (Figure 1C). Since colony formation ability is regarded as an important characteristic of tumor growth *in vitro*, we examined the effect of ORAOV1 silencing on HeLa cell colony formation ability. According to plate colony assay, the colony formation ability were obviously suppressed in the ORAOV1 silenced HeLa cells, and its relative colony formation efficiency decreased to 5-10% of that of the control ones (p < 0.001) (Figure 1D).

### ORAOV1 silencing induces S-phase cell cycle arrest in HeLa cells

**Figure 2:**
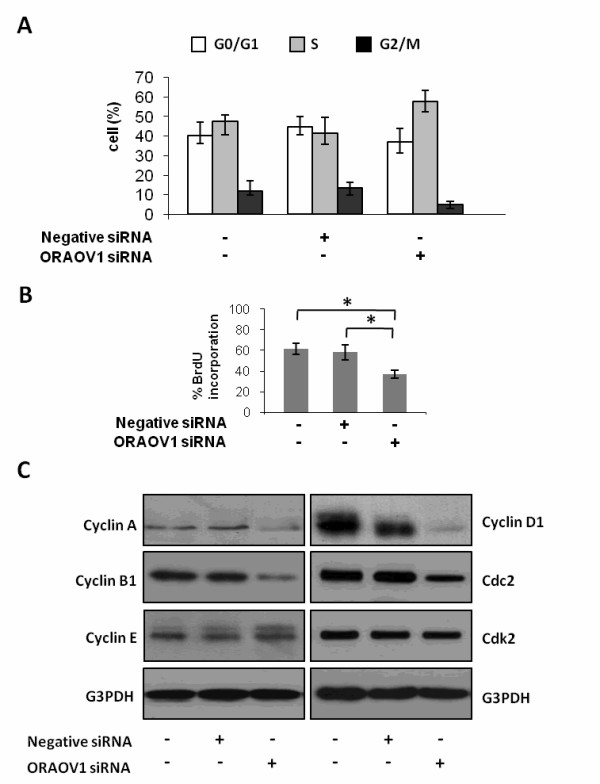
**ORAOV1 silencing induces S-phase cell cycle arrest in HeLa cells**. (A) Quantification of cell cycle distribution determined by flow cytometry analysis at 48 hours posterior to the treatment. Data were Mean ± SE for triplicate experiments. (B) BrdU incorporation assay was performed at 48 hrs posterior to the treatment. Each column represents a mean value of triplicate experiments in each group. Data are Mean ± SE, *, *P *< 0.05. (C) Western blot analyses of the expression of Cyclin A, Cyclin B1, Cyclin E, Cyclin D1, Cdc2, and Cdk2. G3PDH was used as an internal control for equal loading. Data were representative blots from three independent experiments.

To study the potential mechanisms by which ORAOV1 silencing inhibit HeLa cell growth, the effect of ORAOV1 siRNA on cell cycle was evaluated by flow cytometry assay. As shown in Figure 2A, at 48 hours after treatment, the percentages of S-phase cells in blank and negative siRNA-transfected HeLa cells were around 40-50%, whereas that in the ORAOV1 siRNA-transfected HeLa cells were about 60%. In the meantime, 10-15% of blank and negative siRNA-transfected HeLa cells were in G_2_-phase, compared with about 5% in the ORAOV1 siRNA-transfected group. However, no obvious alternation was found in the percentage of G_1_-phase cells in ORAOV1 siRNA-transfected HeLa cells compared with the control. Therefore, ORAOV1 silencing may arrest the cell cycle at the S-phase by inhibiting the S to G_2 _transition in HeLa cells.

Next, we performed BrdU incorporation assay and Western blot assay to investigate the effect of ORAOV1 silencing on DNA synthesis and cell cycle regulators. Our results showed that ORAOV1 silence in HeLa cells caused suppression of DNA synthesis (Figure 2B). In the meantime, a down regulated expression level of Cyclin A, Cyclin B1, Cyclin D1 and Cdc2 was also detected (Figure 2C). These results indicated that ORAOV1 silencing may regulate the cell cycle in HeLa cells by regulating DNA synthesis and the expression of several cell cycle regulators.

### ORAOV1 silencing induces apoptosis in HeLa cells

**Figure 3:**
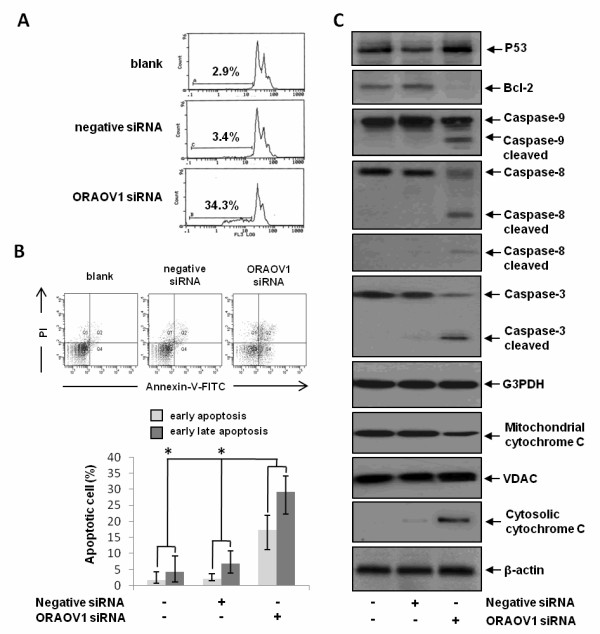
**ORAOV1 silencing induces apoptosis in HeLa cells**. (A) Analysis of HeLa cell apoptosis (sub-G1 cells) upon ORAOV1 silencing by flow cytometry assay using PI staining. (B) Quantitative analysis of cell apoptosis by Annexin V and PI double staining. Data were Mean ± SE for triplicate experiments, *, *P *< 0.05. (C) The effect of ORAOV1 silencing on the expression of P53, Bcl-2, Caspase-3, 8 and 9, and cytochrome c (mitochondrial and cytosolic). G3PDH was used as an internal control for protein equal loading, and VDAC and β-actin was used as equal mitochondria and cytosol loading control respectively. Data was representative blots of three independent experiments.

To study whether the ORAOV1 silencing induced HeLa cell growth inhibition was related to cell apoptosis, the effect of ORAOV1 siRNA on cell apoptosis was evaluated by flow cytometry using PI staining and Annexin-V & PI double staining. As shown in Figure 3A, the percentage of cells in the pre-G1 (apoptotic) fraction in blank, negative siRNA-transfected, and ORAOV1 siRNA-transfected HeLa cells were 2.9%, 3.4%, and 34.3%, respectively. Similar results had been got by Annexin-V & PI double staining. As seen in Figure 3B, the apoptosis rate (Annexin-V +/PI - and Annexin-V +/PI +) of the blank, negative siRNA-transfected, and ORAOV1 siRNA-transfected HeLa cells were about 5%, 5%, and 30%, respectively. Together, these data suggested that ORAOV1 silencing might inhibit the growth of HeLa cells through initiating cell apoptosis.

It is known that apoptosis could be initiated through two signal pathways; the extrinsic and intrinsic pathways [12,13]. We sought to determine which pathway did ORAOV1 silencing lead to activate by examining the important regulators in these two signal pathways. As shown in Figure 3C, the protein level of P53 was up-regulated by ORAOV1 silencing, while Bcl-2 was markedly down-regulated. In the meantime, the protein level of cytochrome c in mitochondria were detected to decrease accompanied by its increase in the cytoplasm. In addition, Caspase 8, 9 and 3 were all found to be activated upon ORAOV1 silence. These data suggested that ORAOV1 silencing could activate cell apoptosis in HeLa cells through extrinsic and intrinsic pathways.

### ORAOV1 silencing suppresses the growth of HeLa cell xenografts in vivo

**Figure 4:**
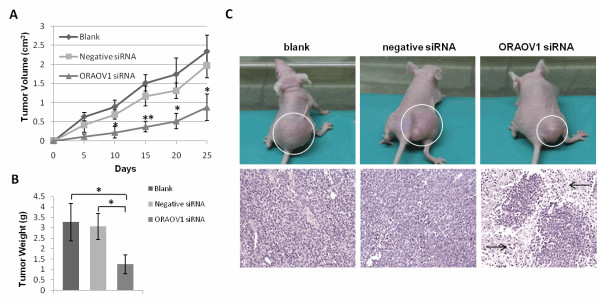
**ORAOV1 silencing suppresses the growth of HeLa cell xenografts *in vivo***. (A) The tumor volume curves. Data were the Means ± SE (n = 5 tumors), *, P < 0.05; **, P < 0.01. (B) The final tumor weight at necropsy 25 days after seeding. Data were the Means ± SE (n = 5 tumors), *, P < 0.05. (C) ORAOV1 silencing induces necrosis in HeLa xenograft tumor tissues. (a) The representative HeLa cell xenografts of each group. (b) Paraffin-embedded sections of representative HeLa xenografts were analyzed by HE staining. Arrows indicate necrosis in tumor tissues.

To test whether the ORAOV1 silencing can affect the growth of HeLa cells *in vivo*, the tumor formation assay was performed as described in Materials and Methods. As shown in Figure 4A, the growth rate of xenografts in ORAOV1 siRNA group was slower than that of the control groups, especially in the first 20 days. The reduction of average tumor volume in the ORAOV1 siRNA group reached about 60-70% compared with the control groups. Moreover, at the end of the experiment, the average tumor weight of the excised tumors from ORAOV1 siRNA group decreased to about 40% of the control groups (Figure 4B). Furthermore, according to the results of HE staining (Figure 4C), necrosis occurred in most of the ORAOV1 siRNA group xenografts in the process of tumor formation.

## Discussion

Cervical cancer is the second most deadly gynecologic malignancy in the world [14,15]. Although it is now regarded as a potentially preventable disease, there is high risk of recurrence and the poor survival rate make a compelling need to explore novel therapeutic targets for its management [16]. It is now well known that the progression of all cancers is characterized by increased-cell proliferation and decreased-apoptosis. Thus, searching for molecular regulators of tumor cell growth has been an important goal of cancer studies. In this study, we present novel evidence that ORAOV1 plays an important role in the regulation of cell growth in cervical cancer HeLa cells. ORAOV1 silence in HeLa cells induces S phase cell cycle arrest by suppression DNA synthesis and regulating the expression of several cell cycle regulators, such as Cyclin A, Cyclin B1 and Cdc2. Moreover, it triggers cell apoptosis in HeLa cells by activating both intrinsic and extrinsic pathways. Interestingly, ORAOV1 is also found to regulate the growth of HeLa cells probably through its effect on Cyclin D1, which is a pivotal regulator in the tumorigenesis of cervical cancer [17,18]. Since all these findings are observed in 11q13 amplified HeLa cells, further studies in other cervical cancer cell lines are still needed to demonstrate the vital functions of ORAOV1 in cervical cancers.

Cell cycle progression is strictly regulated by a series of Cyclins and Cyclin-dependent kinases (CDKs). The Cyclin-CDK complexes act as checkpoints at certain cell cycle phase transition. In eukaryotic cells, checkpoints secure the fidelity of chromosome transmission. When DNA damage or incomplete DNA replication happens, checkpoints will respond by inhibiting CDKs. According to previous studies, Cyclin A, Cyclin E, Cdc2 and Cdk2 are important regulators in the cell cycle transition from S to G_2 _phase [19-21]. Moreover, in association with Cdk2, Cyclin A and E are the major regulators of the activity of Cdc 25B and Cdc 25C, which play important roles in the S phase and G_2_/M phase [22]. For S-to-G_2 _transition, both complexes of Cdc2-Cyclin A and Cdc2-Cyclin B1 act as vital regulators [20,23]. In our study, DNA synthesis was found to be inhibited upon ORAOV1 silence. In the meantime, S phase cell cycle arrest was also detected. Therefore, it may be deduced that ORAOV1 silence in HeLa cells induces suppression of DNA synthesis, which in result activates corresponding cell cycle checkpoints, and induces decrease of the expression of Cyclin A, Cyclin B1 and Cdc2. All these changes lead to a distinct down regulation of the composition of Cdk2-Cyclin A, Cdc2-Cyclin A and Cdc2-Cyclin B1 complexes, which may active the intra-S phase checkpoint and in result cause cells to arrest at S-phase. In previous study about the functions of ORAOV1 in OSCC, ORAOV1 was found to induce S-phase arrest in OSCC cells [4]. In the present study, we want to determine if ORAOV1 is also involved in the regulation of cell cycle progression in cervical cancer cells. Our data clearly showed that S phase cell cycle arrest occurred in ORAOV1-silenced HeLa cells with a suppression of DNA synthesis and a decrease in the expression of several cell cycle regulators such as Cyclin A, Cyclin B1 and Cdc2.

Apoptosis is a complex, multistage, and many genes involved process. Now, it has been understood to be triggered by two distinct signaling pathways [24-27]. One is the death receptor pathway, regarded as the extrinsic pathway; and the other is the mitochondrial pathway, regarded as the intrinsic pathway. For extrinsic pathway, the apoptotic cell death can be triggered from the outside of cells by activating death receptors. Then, through their ligands, such as TNF-α and Fas L, the initiator Caspase 8 and 10 are cleaved and activated, which lead to the motivation of their downstream effector Caspases, such as Caspase 3, 6, and 7 to kill the cell. For intrinsic pathway, apoptosis is mediated by the release of cytochrome c from the mitochondria, which promotes the activation of procaspase-9 into its activated form Caspase 9, and activates the downstream effector Caspase 3, 6, and 7 to trigger cell death. In the intrinsic apoptotic pathway, P53 is proved to promote cell apoptosis through promoting cytochrome c release; while Bcl-2, a member of the Bcl-2 family proteins, has an anti-apoptotic effect by prevent the release of cytochrome c from the mitochondria. In the present study, ORAOV1 silencing was found to induce apoptosis in cervical cancer HeLa cells. Further functional studies showed that ORAOV1 silencing in HeLa cells could increase P53 expression and decrease the Bcl-2 protein level. Furthermore, the expression level of cytochrome c in cytosol was found to be increased with its decrease in the mitochondria, which means the releasing of cytochrome c from the mitochondria. Then, with the activation of the downstream effectors, Caspase 9 and 3, intrinsic apoptotic pathway is determined to be activated by ORAOV1 silencing in HeLa cells. Besides the activation of intrinsic pathway, ORAOV1 silencing was also found to active apoptosis through the extrinsic pathway by inducing Caspase 8 and 3 cleavage and activation. Therefore, ORAOV1 silencing was found to trigger apoptosis in HeLa cells through both pathways, and ORAOV1 is regarded to take a crucial part in the regulation of HeLa cell growth through its roles in cell apoptosis regulation.

Cyclin D1 is an important protooncogene. Together with its corresponding binding partner cyclin-dependent kinases, CDK4 and CKD6, Cyclin D1 acts as a crucial cell cycle regulator which has been regarded to take an important part in the development and progression of several cancers [28]. According to clinical sample studies, Cyclin D1 overexpression has been found in a variety of carcinomas, and have been linked to the early onset, progression and metastasis of tumors [29]. For cervical cancers, the prognostic value of Cyclin D1 was supported by the tight correlation between its overexpression and the clinical grade of cervical cancer [30,31]. In the present study, we found another important gene for cervical cancer tumorigenesis, ORAOV1. Interestingly, according to the mapping results of chromosome 11q13, the distance between CCND1 and ORAOV1 was less than 12 kb [1]. Given the functional roles of Cyclin D1 and ORAOV1 in cervical cancer tumorigenesis, the close proximity of these two genes on chromosome 11q13, it is interesting to determine whether there are interactions exist between these two genes. According to our study, ORAOV1 specifically knocked down by RNAi obviously suppressed the expression of Cyclin D1, which suggests that Cyclin D1 may be a downstream effector of ORAOV1, and ORAOV1 probably takes a part in the regulation of cell growth in cervical cancer cells, in some degree, through its effect on Cyclin D1.

In the present study, ORAOV1 silencing is determined to inhibit HeLa cell growth *in vitro*. Thus, it is of great interest to investigate whether it has similar effect *in vivo*. Through tumor formation assay, ORAOV1 knockdown is demonstrated to suppress the growth of HeLa xenografts. Moreover, by HE staining, necrosis was found in ORAOV1-silenced HeLa xenografts. Since ORAOV1 silencing is found to induce cell cycle arrest and cell apoptosis in HeLa cells, it can be deduced that the necrosis in HeLa cell xenografts may be a result of cell apoptosis induced by ORAOV1 knockdown.

Since the expression of ORAOV1 has been detected in SCCs of different origins, its functional roles in tumorigenesis have been determined in these SCCs [1-4], it is reasonable to speculate that ORAOV1 may be a common regulator in the tumorigenesis of SCCs, and a general therapeutic target for the treatments. Moreover, since two different splice variants of ORAOV1 have been identified [32], the functions of ORAOV1 proteins in SCCs seem to be complicated. Still, a large amount of further studies are needed to clarify the biological functions of ORAOV1 protein family in SCCs, as the biological functions of each ORAOV1 splice variant remains unclear.

## Conclusion

In conclusion, we provide the first evidence that ORAOV1 participates in the regulation of cervical cancer HeLa cell growth through its effect on the cell cycle and apoptosis. Thus, it may be a valuable protooncogene and therapeutic target for cervical cancer management. Moreover, since its functional roles have been determined in tumorigenesis of various SCCs, ORAOV1 is speculated to be a common regulator in SCCs tumorigenesis, and a general therapeutic target for their treatments.

## Materials and methods

### Preparation of siRNA and transfection

ORAOV1 siRNA (ON-TARGET plus SMARTpool, Thermo Scientific Dharmacon) directed against ORAOV1 (GenBank accession no. NM_153451) was used to silence ORAOV1 in the present study. Non-Targeting siRNA (ON-TARGET plus Control siRNA, Thermo Scientific Dharmacon) was used as the negative control. For siRNA transfection, transfection agent DharmaFECT 1 (Thermo Scientific Dharmacon) was used and the transfection was performed following the manufacturer's protocol.

### Cell culture

HeLa cells were obtained from the State Key Laboratory of Biotherapy and Cancer Center at Sichuan University with its identity verified by the short tandem repeat (STR) analysis (see additional file 1). In our study, HeLa cells were cultured in DMEM (Gibco RL, Grand Island, NY) with 10% fetal bovine serum (FBS; Gibco), 100 units/ml penicillin and 100 μg/ml streptomycin, and incubated in a humidified 37°C incubator with 5% CO2.

### Real-time RT-PCR analysis

Total RNA of each sample was isolated from cells by RNeasy mini kit (Qiagen). The mRNA expression level of ORAOV1 was detected using a quantitative 2-step RT-PCR assay with ORAOV1 gene specific primers (forward primer: CCCGCGUGCCGUUCUUACC; reverse primer: CCGGCAGCUUCAGGCACAAAUG). The RT was performed using Thermoscript RT-PCR system (Invitrigen), and the real time PCR was performed using SYBR Green PCR Master Mix (Applied Biosystems) with the ABI 7900HT Sequence Detection Systems. The relative mRNA expression level was determined using the 2-delta delta Ct analysis method, where actin was used as an internal reference.

### MTT assay and plate colony assay

For MTT assay, HeLa cells were plated in 96-well plates at a density of 1 × 10^4 ^cells per well. After transfection for 96 hours, 10 μl MTT with 5 mg/ml concentration was added to each well and cultured at 37°C for 4 hours. Then, the medium was discarded and 100 μl DMSO was added to each well. After incubating for 10 minutes, the absorbance of each well was read by a microplate reader (Model 550, Bio-Rad, Richmond, CA). For plate colony assay, at 24 hours post-treatment, 3 × 10^2 ^HeLa cells of each group were reseeded in each 60 mm tissue culture disk (Falcon) containing DMEM with 10% FBS, respectively, and cultured at 37°C for 2 weeks. The cells were then stained with Giemsa and colonies containing more than 100 cells were counted.

### Cell cycle analysis

48 hours after the treatment, 2 × 10^6 ^HeLa cells of each group were harvested by trypsinization, washed twice with PBS, and fixed with cold 70% ethanol at 4°C overnight. The cells were then washed once with PBS, digested by 200 μl RNase (1 mg/ml) at 37°C for 30 minutes, and stained with 800 μl propidium iodide (50 μg/ml) at room temperature for 30 minutes. Cell cycle analysis was done by using EPICS Elite ESP flow cytometrer (USA).

### Apoptosis assay

Apoptotic cell death was assessed by flow cytometry assay using propidium iodide (PI) staining and Annexin-V fluorescein isothiocyanate (FITC) and PI double staining. The flow cytometry assay using PI staining was performed as described in the part of cell cycle analysis. Annexin-V & PI double staining was performed using Annexin-V FITC Apoptosis Detection Kit (R&D systems, Abingdon, UK) following the manufacturer's protocol. In brief, at 36 hours after the treatment, 2 × 10^5 ^trypsinized cells of each group were stained and analyzed by a flow cytometrer (BD FACSAria™).

### Bromodeoxyuridine (BrdU) incorporation assay

BrdU incorporation assay was performed as described elsewhere [33]. In brief, 48 hrs post treatments; cells were labeled with BrdU (BD Pharmigen) for 1 hour and fixed with 70% ethanol. Then, cells were stained with anti-BrdU antibody and incubated with fluorescein isothiocyanate (FITC)-conjugated secondary antibody. The incorporation of BrdU was measured by fluorescence-activated cell sorting (FACS) analysis with 10,000 cells collected for each assay.

### Western blot analysis

Proteins in each group were isolated and subjected to Western blot analysis as described elsewhere [34]. Isolation of mitochondrial and cytosolic proteins was performed using Mitochondria/cytosol Fractionation Kit (Beyotime Inst. Biotech, Peking, PR China) following the manufacturer's protocol. Antibody to ORAOV1 was from Abcam (AbCam, Cambridge, United Kingdom); antibodies to P53, Caspase-3, Cyclin A, Cyclin D1, Cyclin E, and Cdc2 were obtained from Cell Signaling Technology (Beverly, MA); antibodies to Caspase-9, Bcl-2, and G3PDH were from R&D systems (Abingdon, UK); antibody to Caspase-8 was from Millipore; antibodies to Cdk2, VDAC and β-actin was from Santa Cruz Biotechnology (Santa Cruz, CA), and antibody to cytochrome c was from PharMingen. The secondary antibodies were HRP-linked anti-mouse (7076) or anti-rabbit (7074) IgG from Cell Signaling Technology (Beverly, MA), and anti-goat IgG antibody from Abcam (ab30816). SuperSignal^@ ^West Pico (Pierce) was used to detect the blots.

### In vivo tumor formation assay

All animal studies were conducted following the U.S. Public Health Service's policy on humane care and use of laboratory animals. Fifteen 6-week-old female BALB/c-nu/nu nude mice were purchased from the Animal Center of Sichuan University and randomly divided into three groups of 5 mice each. 1 × 10^6 ^log-growing HeLa cells transfected with ORAOV1 siRNA, negative siRNA, or left untreated were harvested by trypsinization 12 hours after the treatment, washed twice with 1 × PBS, suspended in 100 μl of DMEM without FBS and antibiotics, and injected subcutaneously into the right flank site of each mouse. In the meantime, silencing of ORAOV1in these cells was confirmed by real-time PCR. All the mice were kept in pathogen-free environments, and the xenografts were measured by caliper every 5 days for 1 month. In this study, tumor volume was calculated by the following formula: tumor volume = 1/2 × (longer diameter) × (shorter diameter)^2^. All the mice were sacrificed after 25 days.

### Hematoxylin-eosin staining

The specimens of HeLa xenografts were fixed in 10% buffered formalin, processed, and embedded in paraffin. Sections were cut at 3 μm thickness and stained with hematoxylin and eosin. The slides were examined with Imager.Z1 microscope (Carl Zeiss), and all the micrographs were taken with an AxioCam MRc5 camera (Carl Zeiss).

### Statistical analysis

Statistical analysis was performed with a single-tailed unpaired Student's t-test. Differences with P < 0.05 were considered statistically significant.

## Abbreviations

BrdU: bromodeoxyuridine; CGH: comparative genomic hybridization; G3PDH: glyceraldehyde-3-phosphate dehydrogenase; ORAOV1: homo sapiens oral cancer overexpressed 1; OSCC: oral squamous cell carcinoma; RT-PCR: reverse transcription-polymerase chain reaction; SCC: squamous cell carcinoma; siRNA: small interfering RNA; STR: short tandem repeat.

## Competing interests

The authors declare that they have no competing interests.

## Authors' contributions

LJ designed and participated in the experiments and drafted the manuscript. XZ and ZW carried out the experiments with cells and involved in revision of the manuscript. NJ, YZ and XTL carried out the *in vivo *experiments and participated in the STR analysis. QMC supervised the entire project and involved in revision and final approval of the manuscript. All authors read and approved the manuscript.

## Supplementary Material

Additional file 1 **STR profiles of HeLa cells**. The STR profiles of HeLa cells analyzed by STR profile multiplex system PowerPlex^® ^16 kit (Promega). Click here for file
